# Prevalence and cardiac phenotype of patients with a phospholamban mutation

**DOI:** 10.1007/s12471-018-1211-4

**Published:** 2018-12-13

**Authors:** I. E. Hof, J. F. van der Heijden, E. G. Kranias, D. Sanoudou, R. A. de Boer, J. P. van Tintelen, P. A. van der Zwaag, P. A. Doevendans

**Affiliations:** 1 Department of Cardiology, University Medical Center Utrecht, Utrecht, The Netherlands; 2 Department of Pharmacology and Cell Biophysics, University of Cincinnati College of Medicine, Cincinnati, OH USA; 3 Department of Molecular Biology, Center of Basic Research, Biomedical Research Foundation of the Academy of Athens, Athens, Greece; 4 Department of Cardiology, University Medical Center Groningen, Groningen, The Netherlands; 5 Department of Clinical Genetics, Academic Medical Center, Amsterdam, The Netherlands; 6 Department of Clinical Genetics, University Medical Center Groningen, Groningen, The Netherlands; 7 Department of Cardiology, Central Military Hospital, Utrecht, The Netherlands; 8 Netherlands Heart Institute, Utrecht, The Netherlands

**Keywords:** Phospholamban, Arrhythmogenic cardiomyopathy, Dilated cardiomyopathy, Phenotype

## Abstract

Pathogenic mutations in the phospholamban (*PLN*) gene may give rise to inherited cardiomyopathies due to its role in calcium homeostasis. Several *PLN* mutations have been identified, with the R14del mutation being the most prevalent cardiomyopathy-related mutation in the Netherlands. It is present in patients diagnosed with arrhythmogenic cardiomyopathy as well as dilated cardiomyopathy. Awareness of the phenotype of this *PLN* mutation is of great importance, since many carriers remain to be identified. Patients with the R14del mutation are characterised by older age at onset, low-voltage electrocardiograms and a high frequency of ventricular arrhythmias. Additionally, these patients have a poor prognosis often with left ventricular dysfunction and early-onset heart failure. Therefore, when there is a suspicion of a *PLN* mutation, cardiac and genetic screening is strongly recommended.

## Phospholamban

Pathogenic mutations in the phospholamban (*PLN*) gene may cause inherited cardiomyopathies due to the role of PLN in calcium homeostasis [1, 2]. PLN has a key role in the function of the sarcoplasmic reticulum (SR) which, in turn, is responsible for the distribution and storage of calcium. During systole, calcium is released from the SR into the cytosol, facilitating contraction of the myocyte. During diastole, calcium must be transported from the cytosol back into the SR as an essential step for cardiac relaxation. This transport is enabled by the sarcoplasmic reticulum Ca^2+^-ATPase pump (SERCA2a), which is regulated by PLN [1, 3]. In its dephosphorylated form, PLN interacts with SERCA2a inhibiting calcium transport. Upon phosphorylation of PLN, its inhibitory effect on SERCA2a is relieved and calcium storage in the SR is increased [4, 5]. The activity of SERCA2a and its interaction with PLN determines the rate of relaxation and contraction of the cardiac myocyte [1].

## Phospholamban mutations

Several mutations have been identified in the *PLN* gene in heart failure patients. The first mutation described in the *PLN* gene was an arginine to cysteine missense mutation at residue 9 and was associated with decreased PLN phosphorylation, leading to dilated cardiomyopathy (DCM) in a large American family [6]. Another mutation involved the conversion of leucine at position 39 to a premature stop codon, which was responsible for the development of heart failure in two large Greek families [7]. A third reported mutation was a deletion of amino acid 14 (R14del) and was first described in a large Greek family [8]. Shortly thereafter, this mutation was also identified in an American and a German family [9, 10].

**Fig. 1:**
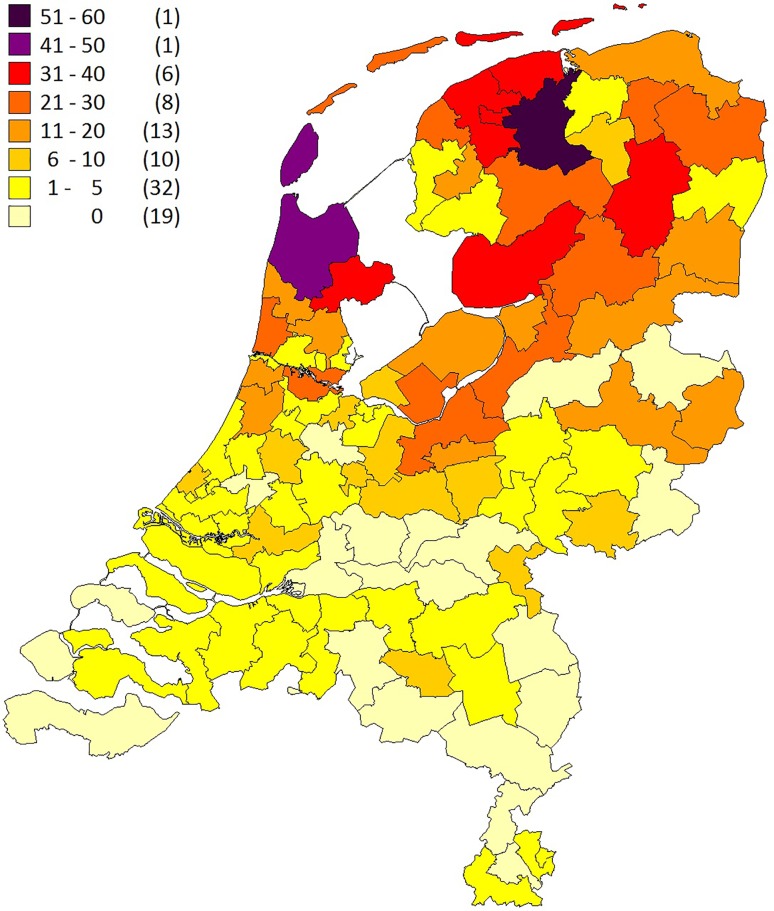
Postal code map showing the distribution of *PLN* p.Arg14del mutation carriers in the Netherlands. The number of *PLN* R14del mutation carriers per region is shown (in parentheses: the number of postal code regions, 90 in total)

Interestingly, the R14del mutation appeared to be the most prevalent cardiomyopathy-related mutation in the Netherlands, being present in 12% of patients diagnosed with arrhythmogenic cardiomyopathy (ACM) and 15% of patients with DCM [2]. All Dutch patients carried the same haplotype, suggesting a founder effect, which was estimated to be between 575 and 825 years old [11]. The geographical origin of this haplotype was found to be in the eastern part of the province of Friesland. Today, over 1000 R14del mutation carriers have been identified and most mutation carriers live in the northern part of the Netherlands, with a gradual decline towards the southern parts (Fig. 1; [11]). Interestingly, a large Spanish family is also known to carry this mutation with the identical haplotype [12].

Examination of a large population-based cohort in the city of Groningen identified 0.07% of those persons (6/8267) to be R14del mutation carriers [13]. Extrapolating this to the whole of the Netherlands, including lower prevalences for the more southern provinces, the total number of R14del mutation carriers may be more than 2000 [11]. In order to identify these unknown mutation carriers, it is crucial to cultivate an awareness of the phenotype of the *PLN* mutation.

## Phenotype

The R14del mutation results in super-inhibition of SERCA2a, which is irreversible. Inhibition of calcium transport and, consequently, cardiac function over years may lead to ventricular remodelling and failure [8]. Additionally, myocardial scarring as well as SR calcium leak may lead to arrhythmias [14]. Consequently, *PLN* mutation may give rise to clinical features of DCM as well as ACM. However, the mutation has also been found in asymptomatic individuals. This may render their identification challenging. Several studies have examined patients with *PLN* mutations and their family members in order to identify specific phenotypic characteristics that distinguish them from other patients with DCM or ACM. Their findings are summarised below.

In patients with the *PLN* R14del mutation, the onset of the disease appears to be age-dependent with a slightly higher frequency in males [15]. Symptoms develop most often in the fifth decade with a mean age at presentation ranging from 40 to 48 years [2, 15, 16]. However, sudden cardiac death may occur earlier in life and has been reported in patients younger than 30 years old [2].

**Fig. 2:**
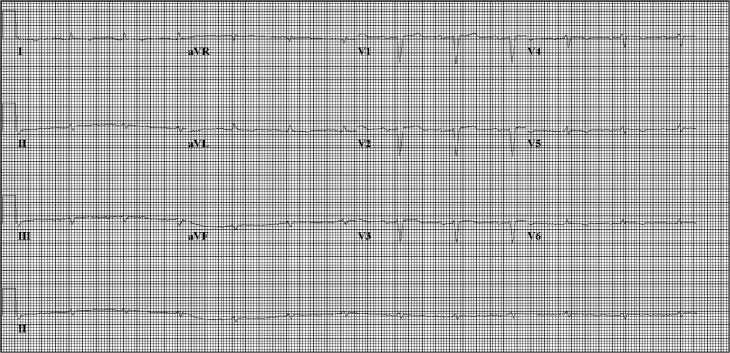
Electrocardiogram of a patient with the *PLN* R14del mutation, with low voltages in the standard leads and negative T waves in left precordial leads V4 to V6

A striking discovery is that many patients with a *PLN* mutation exhibit similar abnormal electrocardiographic characteristics. Most members of the Greek family in which the *PLN* R14del mutation was first described had low QRS complex potentials and decreased R‑wave amplitude [8]. This finding was reproduced in a German family where low R‑wave amplitudes were found in all adult *PLN* R14del mutation carriers regardless of echocardiographic abnormalities [10]. Similarly, within our Dutch population, *PLN* mutation carriers frequently show low-voltage electrocardiograms (ECGs) and, additionally, negative T waves in left precordial leads ([2, 17]; Fig. 2). These features were not observed in non-mutation carriers, which indicates a mutation-associated phenotype [10]. This may be the most striking characteristic and should raise suspicion of a *PLN* mutation, including taking a family history and considering genetic counselling. The substrate for these low-voltage ECGs may be the presence of cardiac fibrosis, which was a frequent finding at histological examination [8, 18].

Patients with a *PLN* mutation demonstrate an arrhythmogenic phenotype. This is reflected by a high rate of positive family history for sudden cardiac death below the age of 50 years and frequent ventricular extrasystoles during Holter monitoring [2, 16, 17]. Additionally, DCM patients with a *PLN* mutation present with ventricular arrhythmias and experience appropriate implantable cardioverter defibrillator (ICD) therapy more often than DCM patients without a *PLN* mutation [2]. In one study, the authors evaluated possible risk factors for malignant ventricular arrhythmias in a large cohort of individuals carrying the *PLN* R14del mutation. They found left ventricular (LV) ejection fraction <45% and (non-)sustained ventricular tachycardia (VT) to be independent predictors for malignant ventricular arrhythmias [15]. These events may precede end-stage heart failure.

Within the subgroup of patients fulfilling the criteria for ACM, patients with a *PLN* mutation have a higher frequency of LV structural and functional abnormalities [16, 17]. Compared to other mutation carriers, they show the most pronounced diminished LV function as determined by echocardiography and cardiac magnetic resonance imaging (MRI) [16, 19]. Furthermore, cardiac MRI scanning exhibits a typical pattern of scarring in the inferolateral LV wall, which is likely reflected by the negative T waves in the inferolateral ECG leads. Autopsy findings have corroborated the presence of extensive fibrosis, which may represent re-entry circuits that likely contribute to malignant arrhythmias.

Finally, the *PLN* R14del mutation may be associated with a poor prognosis. In the Greek family, the mutation was associated with DCM and death by middle age [8]. In the German family none of the mutation carriers survived beyond 50 years of age [10]. Similar findings were found in a large Dutch population where mortality rates were increased compared to those in patients without the *PLN* mutation, mainly between the ages of 25 and 74 years [15].

## Treatment

Currently, there are no clinical studies regarding the treatment of patients with a *PLN* mutation. It is therefore recommended that the current guidelines on heart failure and prevention of sudden cardiac death (sections on DCM and ACM) be applied to determine the optimal medical treatment, exercise restriction, and whether VT ablation or ICD implantation is indicated [20, 21]. In addition, referral to a centre offering clinical genetics services is recommended.

At present, a Dutch study is randomising asymptomatic *PLN* mutation carriers to treatment with eplerenone versus placebo to determine if eplerenone will slow down progression of the disease (iPhorecast study). We are awaiting the results.

For patients as well as their doctors a *PLN* foundation has been established that provides information on new developments (https://hartspierziektepln.nl).

## Conclusion

In the Netherlands the *PLN* R14del mutation is one of the most prevalent cardiomyopathy-related mutations. As a founder mutation its origin has been traced to the northern parts of the Netherlands. *PLN* mutation carriers have a highly variable phenotype, which ranges from asymptomatic to cardiomyopathic, including clinical features of ACM as well as DCM. The most striking characteristic is the low-voltage ECGs. In addition, patients with a *PLN* mutation are characterised by a late onset of symptoms, an arrhythmogenic phenotype, a higher frequency of LV dysfunction and a poor prognosis. Therefore, upon suspicion of a *PLN* mutation, referral to a centre offering clinical genetics services and cardiac and genetic screening is strongly recommended.
